# The Impact of Social Entrepreneurship, Corporate Social Responsibilities, and Working Capital Management Practices on the Performance of Tourism Small–Medium Enterprises (SMEs) During COVID-19: Moderating Role of Employee Behavior

**DOI:** 10.3389/fpsyg.2022.869856

**Published:** 2022-05-23

**Authors:** Yuanyuan Li, Israr Ahmad, Hassan Raza, Anusara Sawangchai, Edwin Ramirez-Asis, Edwin Asnate-Salazar

**Affiliations:** ^1^Department of Tourism Management, Business School, Xuzhou University of Technology, Xuzhou, China; ^2^School of Business Management, Universiti Utara Malaysia, Sintok, Malaysia; ^3^Faculty of Business and Management, Universiti Teknologi Mara Malaysia, Sarawak, Malaysia; ^4^Business Administration in Entrepreneurship Program, Faculty of Management Sciences, Phuket Rajabhat University, Phuket, Thailand; ^5^Department of Business Science, Universidad Señor de Sipán, Chiclayo, Peru; ^6^Department of Science, Universidad Nacional Santiago Antunez de Mayolo, Huaraz, Peru

**Keywords:** corporate social responsibilities, social entrepreneurship, working capital management practices, the performance of SMEs, employee behavior

## Abstract

The failure or success of an enterprise depends upon its working capital management practices (WCMP) along with effective corporate social responsibilities (CSR) and social entrepreneurship (SE). These factors ensure not only the soundness of financial indicators but also the profitability of an enterprise. Therefore, the objective of this study was to determine the impact of CSR, SE, and WCMP on the performance of small–medium enterprises (SMEs) of tourism sector, during the lockdown period in Malaysia. The goals also include the investigation of moderating role of employee behavior (EB) among the nexus of CSR, SE, WCMP, and performance of SMEs. The survey was performed to find out the practices adopted by the SMEs during corona days. A quantitative research method has been adopted to get data with convenient sampling technique, and PLS-SEM has been exploited to find out the significant nexus among CSR, SE, WCMP, and the performance of SMEs. The results indicate that CSR, SE, and WCMP have a positive association with the performance of SMEs in Malaysia. The results also indicate that EB positively moderates among the nexus of CSR, SE, WCMP, and the performance of SMEs in Malaysia. Stock review strategies by the SMEs during the corona days also exhibit significant differences except for no stock review. Significant differences between the enterprises show a lack of adaptation of the financial indicators that determine enterprise soundness. The study not only provides guidelines for entrepreneurs of SMEs but also helps in maintaining standards for the evaluation of the enterprises.

## Introduction

Business involves the individual struggle as well as the social approach that has been constructed by entrepreneurs during COVID-19. This approach which helps sustainable organizations also induces its impact to enhance the performance in Malaysian SMEs. Therefore, social entrepreneurship (SE) in an organization can establish considerable growth, which could explore the significant performance in SMEs (Bojica et al., 2018). Organizational performance is not only prevalent in SE, but many other factors also induce a significant role during COVID-19. For sustainability in organizations, SE induces its practices to attain better performance in SMEs. In many Malaysian SMEs, SE is countered as important involvement that contributes significant performance. This is the establishment of sustainable SE *via* different networks in some countries (Jayakar Pai and More, 2018). A variety of supporting networks through SE help in the performance of SMEs to strengthen ties in COVID-19. In many industries, corporate social responsibility inserts an eminent role in the upbringing of organizational performance. But the SMEs’ performance has probably achieved the role of corporate social responsibilities (CSR) in many countries, including Malaysia. This role has positively depicted the voluntary bargaining effects on the performance of some industries (Haslam, 2018; Ajaz et al., 2020). Therefore, the regulatory requirements are also focused on considering some compliance elements prevailing in governing structure (Abdullah et al., 2018a).

The objective of the ongoing research was to examine the impact of CSR, SE, and working capital management practices (WCMP) on the performance of SMEs along with the investigation of moderating role of employee behavior (EB) among the nexus of CSR, SE, WCMP, and performance of SMEs during the lockdown period in Malaysia. The survey was performed to find out the practices adopted by the SMEs during corona days.

**FIGURE 1:**
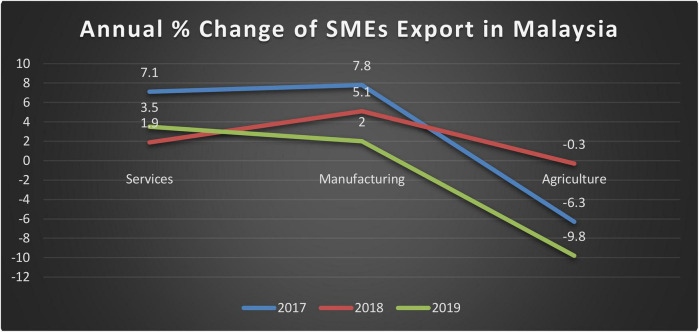
Annual % change in SMEs export in Malaysia.

There is an inclusion of corporate social responsibility in the organizational structure. This benefits the organizations through variant means which includes performance in most of the organizations during COVID-19. The COVID-19 affects the safety and health of the people (Hao et al., 2020) along with business activities. Usually, in Malaysia, corporate social responsibility is significantly emphasized in the context of competitive markets. This ultimately affects the choices of brands by customers and the responses of customers (Han et al., 2020). SMEs are viewed with some significant rise in the last few years when the SMEs have been highlighted internationally. The GDP of SMEs has grown by about 5.8% in 2019 compared with last year at 6.2% in 2018. The GDP of SMEs has been bifurcated according to the registered and non-registered businesses. Therefore, some non-SMEs have been registered at 4.3% in 2019, which has shown a significant increase by comparing increment of 2018 at 3.4%. SMEs have also contributed a significant portion to the GDP during 2018–2019. This increment has depicted a significant rise of 38.9%, which is 0.06% enhanced from 2018 at 38.3%. This depiction has added the prices that have been changed to 552.3 billion in 2019, comparing 522.1 billion during 2018. With the probable effects of corporate social responsibility, the performance of SMEs has attained a considerable rise. Therefore, with the inclusion of some working capital management, new opportunities could also enhance the performance. This is usually maintainable in most Malaysian SMEs where the performance has attained a prominent rise. This endorses the significant relationship between corporate governance and working capital management for the assessment of firm performance (Kayani et al., 2019). Annual change in SMEs Export in Malaysia is given in Figure 1. There is a decreasing trend reported in the year 2019. Although there were positive signs in the year 2018 in all three sections, there is a negative trend in 2019.

It is though profitable for the SMEs but also helps to establish governing structure for the higher performance of SMEs. Although the high growth is depicted by some financial decisions during COVID-19 which are mostly relied on the governing bodies, the effectiveness of some dominant elements induces a positive rise among Malaysian SMEs performance. The indulging attribute of working capital management depicts higher growth among the emerging firms in many countries (Boţoc and Anton, 2017). With the emergence of some elected variables, the role of EB is also dominant among SMEs’ performance. This emergence which affects the performance of SMEs also asserts possible influence upon SE, working capital management, and CSR. It also asserts moderating effect on the performance of organizations that are competing with the spiritual values prevailing in helping behaviors of employees (Ahmed et al., 2019). Wisdom is prevalent, a sense of community, and a sense of meaning that could develop significant growth in various sectors.

Complete lockdown during the corona days has changed the working patterns of the enterprises. WCMP are the main factors that determine the survival of an enterprise these days. An organization of current resources and liabilities of entrepreneurship depicts the WCMP. The success of these practices results in decreasing real cash conversion cycles. These practices not only result in improvement in profitability but also provide standards to the investors for evaluation. Subtraction of current liabilities from current assets of an enterprise provides its working capital. It is mostly associated with receivable accounts, cash, delayed accounts, inventory, and payable accounts. The firms have bankruptcy problem due to lack of management practices. A significant level of current resources may decrease the danger of cash by leasing or renting immovable property. But the comparable strategy cannot be followed for the parts of working capital. Nonetheless, assessing working capital needs is an overwhelming job as it varies from business to business, cash flow cycles, and stock accessibility. Application of standards and practices of working capital reduces the danger and improves execution. The concept of entrepreneurship is mostly associated with SMEs by many researchers and policymakers. Unemployment can be decreased by getting benefits from the potential of SMEs. These are considered as an important source of stable economic growth along with new ways of working. The researchers describe various kinds of financial, environmental, and managerial problems associated with SMEs (Abdullah et al., 2018b; Shehzad et al., 2020; Sarfraz et al., 2021a,b).

Most of the researchers link these problems with poor business and working capital management. Working capital management determines the financial soundness of a firm. It also determines the firm profitability. It also points out the firm ability to handle some uneven financial crisis. The keeping up of satisfactory working capital could not be simply addressed. The progression of assets for a business is as important as blood is necessary to keep our bodies alive. Cash and cash flows were the king and queen of the financial management of any firm. The firm’s short-term assets and liabilities describe the working capital. It is necessary to run an everyday business and considered paying employees’ wages before the collection of the revenues. Working capital is considered positive when a company can pay its transient liabilities, while it is considered negative when a company cannot pay its transient liabilities within its current resources. Accordingly, the inability to get ready for expanding working capital needs can prompt genuine income issues. Considering the need for the project, the study aims to find out WCMP by small–medium enterprises (SMEs) during the corona days.

The services in SMEs have recorded significant growth of about 7.4% while inducing the growth of 8.1% in 2018. There is a probable momentum in the SMEs businesses indulged in retail, wholesale, and other sub-sectors that have been grown by 7.6% during 2018. Besides this, the business services have also been grown by 7.7% during 2018 where the products have attained a growth of 4.3% with a considerable rise of 5.9%. Some other sub-sectors which are associated with SMEs have depicted a slower rise of 4.9 and 3.2%, respectively, where the different values are added. SMEs indulged in the mining sector have been enhanced by 4.7% with the relevance of construction by 4.0%. While using the approach of EB, the performance of SMEs could not be omitted. This emergence of the behavioral approach helps to counter a variety of challenges prevailing in the competitive environment during COVID-19. However, the ethical practices of EB are important and the impacts of organizational justice and ethical leadership also fit in the organizational structure (Al Halbusi et al., 2020).

This ethics of business involves a variety of comparing elements that belong to the organizational performance. Therefore, participation is always required to contribute a significant portion to organizational growth (Sarfraz et al., 2018). Certain depths and scopes also hinder in the performance measures which require employee participation and pay for performance (Wang, 2018). This benefits the SMEs from different aspects in collecting momentum of growth. The role of EB is important though, and the business strategies are also important. This involvement refers to the approach of some practicing risks, which are associated with management playing a role in SME’s performance and business strategies (Rehman and Anwar, 2019). Proper indication of SME’s performance is rendered by the facilitation of management practices and corporate structures. The study follows the structure. The first section contains the “Introduction,” and the second section is about the “Literature Review.” The third section contains the “Research Methodology,” the fourth section includes the “Data Analysis,” and the fifth section contains the “Conclusion, Implications, and Limitations.”

## Literature Review

The study based on social learning theory that was introduced by Albert Bandura suggests the social behaviors are learned by observing the others’ behaviors. Furthermore, people can learn the new behaviors and information by observing the other individuals in environmental and social circle. This theory supported the phenomena of this study.

Some aftermaths of SE are dominant with reference to some exclusionary practices during COVID-19. These practices are widely associated with businesses and the events of the great recession which emphasizes the approach of SE. It focuses on the growing emergence of SE, which inserts capable measures to enhance the performance of SMEs. Many Malaysian firms have attained considerable growth with the reflection of SE during COVID-19. This renders the driving change in institutions where SE widely dominates with a variety of capabilities (Warnecke, 2018). The diversification of different elements that are involved in SE involves sex, age, social issues, and size with the leadership characteristics, and the larger challenges are depicted in the variety of firms. The role of SE fortifies the mentors which develop the firm’s performance. SE is a positive association of developing linkages that bring positive change to the SME’s performance in COVID-19. This wide establishment of implications also states the initiatives that are dominant in organizational performance due to significant future directions. This explores the catalyzing situation of SE among the variety of firms in many countries like Malaysia (Mirvis and Googins, 2018). Therefore, it also helps to succeed in the organizational performance and the development of a sustainable environment for the SMEs sector. It is upon the catalyst approach of SE which states the brief inclusion in SMEs performance. This dominates with the wide relationship of corporate social responsibility which provides the stimulus of the firm’s performance in COVID-19. The effects are comprehensive with the efforts that are inserted in the organizations with critical views. Therefore, by combining the efforts over sustainability in organizations, the importance of SE and CSR positively dominates (Pandey et al., 2020). This domination develops the ecosystem which counters challenges and enhances the opportunities. This enhancement is referred toward the supportive opportunities which have material effects on the performance of SMEs.

Different dramatic situations are prominent in the organizational culture. This is due to the changing market environments and wide adaptation of technological approaches. This adaptation provides a variety of opportunities and also states wide challenges mostly for SMEs. This is strategically important for larger organizations and SMEs due to the guided approach of different market combinations during COVID-19. A comprehensive conceptualizing orientation states the supportive aspects of SME’s performance by the emergence of the digital economy (Quinton et al., 2018). While creating the supportive aspects, the hindering and supporting elements are important for evaluating the possible performance of organizations. Wide interlinked establishment of SMEs’ performance is usually stated by the indulged role of SE in COVID-19. This helps to uplift the performance of the SMEs sector in Malaysia and many sectors of other countries. Usually, the innovation has also constructed a positive development process for the SMEs which dedicates the source of knowledge (Hervas-Oliver et al., 2020). This is extracted from the external factors which are associated with SE and SMEs’ performance. A variety of tactics and processing of technologies have linked innovation with higher achievement of SMEs performance.

**H1**: Social entrepreneurship significantly and positively influences SME’s performance in Malaysia.

Many factors have an important role in the organizational hierarchy, and CSR is one of them (Basheer et al., 2020). This enabling element of CSR positively depicts the abilities which have induced considerable growth of SME’s performance. CSR are globally used in many sectors which associates its linkage with the loyalty to companies and its high performance in COVID-19. Therefore, the wide association of corporate social responsibility influences the perception of consumers on global brands also (Crespo and Inacio, 2019). This impact is also supported by the positive measures by the inducement of consumer identification in companies. It helps to retain relationships and to increase the growth of sales and services. Although many competitors also prevail in competitive markets, the corporate association helps to enable the process of consumer identification, especially during COVID-19. This measure reaches the system of education which could be developed in the companies and colleges by developing a sense of responsibility (Wang, 2018). These reforms and new formation could bring a positive result to the organizational performance. Usually, the SMEs could attain considerable growth by playing unique roles of corporate social responsibility. In the exploration of linkage among firms’ performance and the uniqueness of corporate social responsibility, some corresponding elements of CSR have positively illustrated the significant SMEs’ roach toward SMEs’ performance in Malaysia during COVID-19. This also depicts the conducted assessments in many international SMEs which bring eminent change in their performance (Ahmed and Streimikiene, 2021).

The indulging attitude of lining firm competitiveness and corporate social responsibility is described through a variety of dimensions (Lu et al., 2020a). This explanation states the inducement of voluntariness, shareholder involvement, and social and environmental impact on the SMEs’ performance. It also elaborates the impacts that last the consumer efficiency and is beneficial for the uplifting of SMEs’ performance.

The dimensions of corporate social responsibility state the structures which could bring significant performance. These dimensions are positively stated by the relativeness of financial capacity and the demanding needs of consumers in COVID-19 (Khalid et al., 2022). Therefore, economic, social, and environmental elements are positive approaches of corporate social responsibility which states the image of companies. The image overall states the performance of SMEs which is improved by the market agility and supply chain flexibility (Benzidia and Makaoui, 2020). The perspectives of consumer response and organizational positions are effective tools for elaborating the enhancing performance and competitiveness in SMEs, while drawing the resources that state the useful guidelines in SMEs’ performance (Lu et al., 2020). The orientation in an organization is depicted as a positive implication among SMEs. This plays a role not only in the market but also in entrepreneurship with significant enhancement of performance during COVID-19. The usefulness of strategic orientation fits toward the performance of SMEs through different factors with certain access (Park and Seo, 2018). This effectiveness of CSR places orientation and learning abilities that could help independent to enhance the SMEs’ performance.

**H2**: Corporate social responsibilities significantly and positively influence SME’s performance in Malaysia.

Working capital is mostly linked with financial decision making, but the emergence of CSR among SMEs’ performance sustained the benefits. These benefits are probably associated with the working capital management in most of the corporate sectors, especially during COVID-19. These benefits are availed for the companies’ higher profits and the sake of the company’s performance. Therefore, the impact of capital expenditure is also vital among emerging economies with slight financial constraints (Mielcarz et al., 2018). These constraints have a significant accumulation of working capital management which enhances the SME’s performance from various aspects. Certain constraints linked with investment also state reverse effects on the SME’s performance which could be covered by working capital management. The sensitive operations are unpredictable due to the usage of external finance in SMEs. The use of slight forces with the relevance of decision making uplifts the performance of organizations (Malkawi and Khayrullina, 2021). These uplifting events could eliminate the dominance of trade credits due to the circulation of the business cycle in COVID-19. It is transmitted through the counterparts of SMEs, which induces changes with the reduction in some investing methods. It is important for the value enhancement among working capital management that eradicates the financial constraints (Mai et al., 2021). The increment in working capital among private and international SMEs is a positive approach to bring higher performance. However, inventory management is also indulged in the organizations, especially in COVID-19. Therefore, it brings effective policies among the working capital management by establishing management structures under the constraints of working capital (Ionescu, 2021a). The posture of these constraints could be eliminated by the effective use of working capital management which replenishes the external effects. These effects contain all credit and supplier lingering elements that resist the performance of SMEs.

Certain determinants of working capital management place external effects on SMEs’ performance. Usually, in Malaysia, the elements of the socio-economic structure are most relying on factorial effects on the performance of organizations during the peak of COVID-19. This could state various influences whether they belong to the short-term effects or long-term impacts but carries the indicators which are prominent in organizations. Many determinants prevail in the organization that is relative to the SME’s performance and have a significant impact (Cicea et al., 2019). This addresses the performance of SMEs by the inducement of macroeconomic views. The impacts of working capital on SME’s performance are widely stated in the context of external views. Therefore, the dominance of numerous elements has a wide impact on the orientations of entrepreneurship in COVID-19 (Sarfraz et al., 2019). This relationship states the dimensions of performance that are linked with the SMEs of developing countries. Significant interrelationship exists among SME performance, entrepreneurial orientations, and support programs of government (Nakku et al., 2020). This is the preliminary statement by the financial and non-financial perspectives which states the risks, competitiveness, and innovativeness over the performance of SMEs.

**H3**: Working capital management practices significantly and positively influence SME’s performance in Malaysia.

Employee behavior is the moral ownership that is induced by the employee toward companies. This is the sole contribution of employees which asserts its dominance among the SMEs performance. SMEs of Malaysia state the contribution of EB which enlarges the support and ethical context toward performance during COVID-19 (Sawangchai et al., 2022). However, moral ownership has a significant role in the ethical behavior of employees and organizational support (Jino and Dyaram, 2019). Therefore, the extension of institutional differences states the structural modeling which enables the importance of EB moderating role in SMEs. EB states the dominance over SE and the performance of SMEs in COVID-19. This inducement is considered as a lack of sociological interpretations. It develops a considerable role in SE with a wider context of social perspectives of EB (Kovacova and Lewis, 2021). Among the different programs of working communities, SE has a positive understanding of EB (Hopkins and Potcovaru, 2021). This is illustrative of SE which has an eminent role in moderating EB. The critics of corporate social responsibility also assert the moderating effect of EB (Marakova et al., 2021). Through a variety of EB dimensions, the role depicts its possible impact on corporate social responsibility which is associated with SMEs’ performance. Although the moderating role of EB significantly impacts many other factors, the organizational performance has the departing effect on EB. In many sectors, corporate social responsibility has an important role but the emerging effect of EB on it is also operational (Mohammed, 2019).

Management controls are significant measures for the increment in SME’s performance during COVID-19. Therefore, effective incorporations among the EB are dominantly related to the innovations and harnessing creativities. This states the variety of convergence and evolution impacts of quality management on the performance of organizations (Dahlgaard-Park et al., 2018). EB significantly states its impact on working capital management and its linkage with SMEs’ performance. The excellence structure is developed by the moderating role of EB which illustrates the relationship between working capital management and performance (Durana et al., 2021). The performance of organizations is linked with entrepreneurial intentions which are dependent on EB usually during COVID-19. This link is expanded by the umpiring role of orientation structure which is correlated with the significant influence of EB. The moderating role of EB is strengthened with the practices of SMEs performance because of its link with work factors of social structure. Strong linkage prevails among the managerial capability and entrepreneurial orientation by the clear image of SMEs’ performance and social capital (Tijani and Osagie, 2021). Practices of human resources development specify its linkage with EB, while the moderating effect of EB is counterproductive which examines its linkage with the performance of SMEs (Stojanovic et al., 2020). The counterproductive behavior of workers is positively related to the HR development practices where the engagement of employees matters (Kura et al., 2019). This engagement is the underlying relationship between firms’ performance and EB. Therefore, the moderating influence of EB asserts the significance of practices and intentions toward SME’s performance.

**H4**: Employee behavior significantly moderates the relationship between SE and SMEs’ performance in Malaysia.

**H5**: Employee behavior significantly moderates the relationship between CSR and SMEs performance in Malaysia.

**FIGURE 2:**
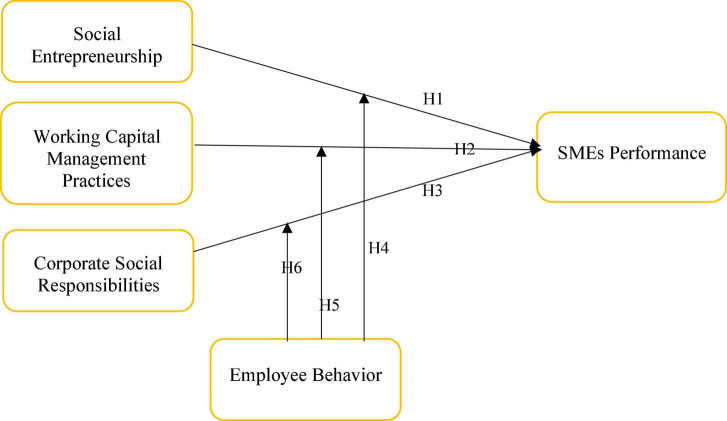
Research framework.

**H6**: Employee behavior significantly and positively moderates the relationship between WCMP and SMEs performance in Malaysia. Figure 2 shows graphical representation of the proposed hypotheses.

## Research Methodology

The objective of the ongoing research was to examine the impact of CSR, SE, and WCMP on the performance of SMEs along with the investigation of moderating role of EB among the nexus of CSR, SE, WCMP, and performance of SMEs during the lockdown period in Malaysia. The survey was performed to find out the practices adopted by the SMEs during corona days. The quantitative research method was adopted to get data from the respondents under positivism philosophy and deductive approach. The data were collected through convenient sampling technique. The employees of SMEs in tourism sector that are currently operating in Malaysia are the respondents and send the surveys by mail and personal visit. A total of 580 surveys were sent by mail and personal visit, and out of them, only 410 were returned, representing a response rate of 70.69 percent.

Moreover, PLS-SEM was used to find out the significant nexus among CSR, SE, WCMP, and the performance of SMEs. The smart-PLS was used because the complexity of the framework along with testing of the hypotheses is the main goal of the study (Hair et al., 2017). Measurement assessment model was used to check the reliability and validity of data, and later on, structural equation model was used to run the regression analysis between constructs.

This study has adopted three predictors named SE that has ten items, CSR that have five items, and WCMP that have eight items (Afrifa, 2016). In addition, the present study has used EB as a moderating variable that has 12 items and performance of small and medium enterprises (SMEP) as a dependent variable that has ten items (Ogunyomi and Bruning, 2016). Some items of constructs are deleted because the cross-loadings of items are less than 0.5 (Boateng et al., 2018).

## Data Analysis

Table 1 shows the profile of respondents. Males are 53%, and females are 47%. Singles are 33%, and married are 67%. Ages between 19 and 30 are 12%, between 31 and 40 are 24%, between 41 and 50 are 25%, between 51 and 60 are 30%, and more than 60 are 10%. Sixteen percent are having intermediate education, 30% bachelor’s degree, 40% master’s degree, and 14% MPhil and others Moreover, executives are 6%, senior managers are 9%, middle managers are 41%, and bottom-line managers are 45%.

**TABLE 1:** Respondents Profile.

Items	Frequency (*N* = 410)	(%)
**Gender**		
Male	218	53
Female	192	47
**Marital status**		
Single	137	33
Married	273	67
**Age**		
19–30	49	12
31–40	97	24
41–50	102	25
51–60	123	30
>60	39	10
**Education**		
Intermediate	67	16
Bachelor	123	30
Master	163	40
MPhil/Others	57	14
**Designation**		
Executives	23	6
Senior managers	36	9
Middle managers	167	41
Bottom-line mangers	184	45

**FIGURE 3:**
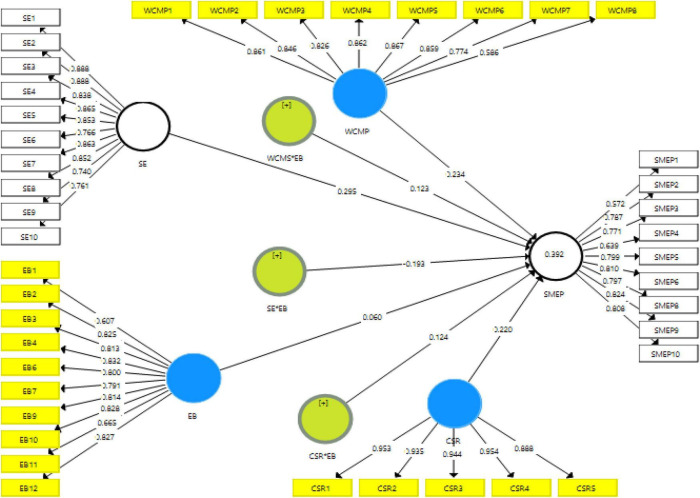
Measurement model assessment.

The present study has examined the convergent validity first to check the measurement model assessment. The convergent validity is related to the relationships among the items, and the outcomes have exposed that the values of composite reliability (CR) along with alpha are higher than 0.70. In addition, the values of loadings along with AVE are more than 0.50. These outcomes have shown valid convergent validity and high correlation among items. These values are highlighted in Table 2. Figure 3 indicates the measurement model assessment.

**TABLE 2:** Convergent validity.

Constructs	Items	Loadings	Alpha	CR	AVE
Corporate social responsibilities	CSR1	0.953	0.964	0.972	0.874
	CSR2	0.935			
	CSR3	0.944			
	CSR4	0.954			
	CSR5	0.888			
Employee behavior	EB1	0.607	0.938	0.940	0.614
	EB10	0.828			
	EB11	0.665			
	EB12	0.827			
	EB2	0.825			
	EB3	0.813			
	EB4	0.832			
	EB6	0.800			
	EB7	0.791			
	EB9	0.814			
Social entrepreneurship	SE1	0.888	0.951	0.958	0.694
	SE10	0.761			
	SE2	0.888			
	SE3	0.838			
	SE4	0.865			
	SE5	0.853			
	SE6	0.766			
	SE7	0.863			
	SE8	0.852			
	SE9	0.740			
SMEs performance	SMEP1	0.572	0.907	0.924	0.579
	SMEP10	0.808			
	SMEP2	0.787			
	SMEP3	0.771			
	SMEP4	0.639			
	SMEP5	0.799			
	SMEP6	0.810			
	SMEP8	0.797			
	SMEP9	0.824			
Working capital management practices	WCMP1	0.861	0.926	0.940	0.664
	WCMP2	0.846			
	WCMP3	0.826			
	WCMP4	0.862			
	WCMP5	0.867			
	WCMP6	0.859			
	WCMP7	0.774			
	WCMP8	0.586			

The present study has also examined the discriminant validity in order to check the measurement model assessment. First, cross-loadings along with Fornell–Larcker have been executed. The discriminant validity is related to the relationships among the variables, and the outcomes have exposed that the values that exposed the nexus with the variable itself are higher than the values that exposed the nexus with other variables. These outcomes have shown valid discriminant validity and low correlation among variables. These values are highlighted in Table 3 and Table 4.

**TABLE 3:** Fornell–Larcker.

	CSR	EB	SE	SMEP	WCMP
CSR	0.935				
EB	0.572	0.784			
SE	0.487	0.285	0.833		
SMEP	0.472	0.311	0.515	0.761	
WCMP	0.422	0.288	0.440	0.423	0.815

**TABLE 4:** Cross-loadings.

	CSR	EB	SE	SMEP	WCMP
CSR1	**0.953**	0.514	0.451	0.457	0.370
CSR2	**0.935**	0.516	0.454	0.415	0.432
CSR3	**0.944**	0.532	0.453	0.443	0.415
CSR4	**0.954**	0.508	0.454	0.460	0.371
CSR5	**0.888**	0.607	0.464	0.427	0.389
EB1	0.288	**0.607**	0.464	0.427	0.389
EB10	0.303	**0.828**	0.127	0.202	0.157
EB11	0.274	**0.665**	0.160	0.262	0.263
EB12	0.305	**0.827**	0.136	0.199	0.163
EB2	0.275	**0.825**	0.142	0.143	0.142
EB3	0.271	**0.813**	0.130	0.105	0.099
EB4	0.281	**0.832**	0.151	0.149	0.148
EB6	0.276	**0.800**	0.134	0.108	0.109
EB7	0.279	**0.791**	0.067	0.111	0.120
EB9	0.256	**0.814**	0.115	0.089	0.106
SE1	0.401	0.225	**0.888**	0.472	0.378
SE10	0.323	0.155	**0.761**	0.304	0.359
SE2	0.391	0.229	**0.888**	0.459	0.379
SE3	0.416	0.264	**0.838**	0.454	0.390
SE4	0.441	0.293	**0.865**	0.466	0.352
SE5	0.452	0.233	**0.853**	0.447	0.379
SE6	0.318	0.164	**0.766**	0.309	0.354
SE7	0.452	0.281	**0.863**	0.464	0.355
SE8	0.447	0.231	**0.852**	0.450	0.372
SE9	0.374	0.260	**0.740**	0.400	0.361
SMEP1	0.361	0.319	0.287	**0.572**	0.196
SMEP10	0.306	0.236	0.375	**0.808**	0.324
SMEP2	0.392	0.262	0.460	**0.787**	0.347
SMEP3	0.432	0.236	0.461	**0.771**	0.369
SMEP4	0.349	0.222	0.255	**0.639**	0.236
SMEP5	0.410	0.260	0.437	**0.799**	0.344
SMEP6	0.303	0.207	0.379	**0.810**	0.315
SMEP8	0.328	0.182	0.393	**0.797**	0.357
SMEP9	0.333	0.220	0.412	**0.824**	0.360
WCMP1	0.352	0.227	0.373	0.352	**0.861**
WCMP2	0.337	0.251	0.356	0.335	**0.846**
WCMP3	0.376	0.266	0.362	0.303	**0.826**
WCMP4	0.364	0.287	0.379	0.322	**0.862**
WCMP5	0.377	0.234	0.400	0.374	**0.867**
WCMP6	0.388	0.242	0.396	0.417	**0.859**
WCMP7	0.341	0.263	0.330	0.359	**0.774**
WCMP8	0.179	0.085	0.248	0.257	**0.586**

*All cross-loading values should be higher than 0.7 these highlighted values are acceptable in table.*

Second, the heterotrait–monotrait ratio has been executed and the outcomes have exposed that the values are lower than 0.85. These outcomes have shown valid discriminant validity and low correlation among variables. These values are highlighted in Table 5.

**TABLE 5:** Heterotrait–monotrait ratio.

	CSR	EB	SE	SMEP	WCMP
**CSR**					
EB	0.449				
SE	0.504	0.212			
SMEP	0.504	0.247	0.539		
WCMP	0.444	0.229	0.469	0.452	

**FIGURE 4:**
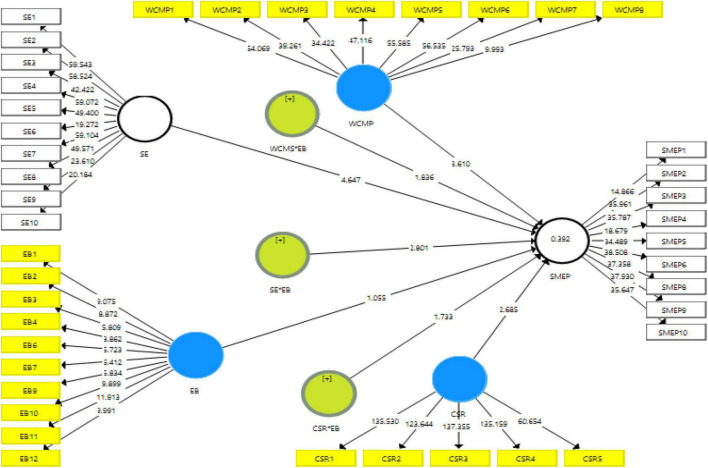
Structural model assessment.

**FIGURE 5:**
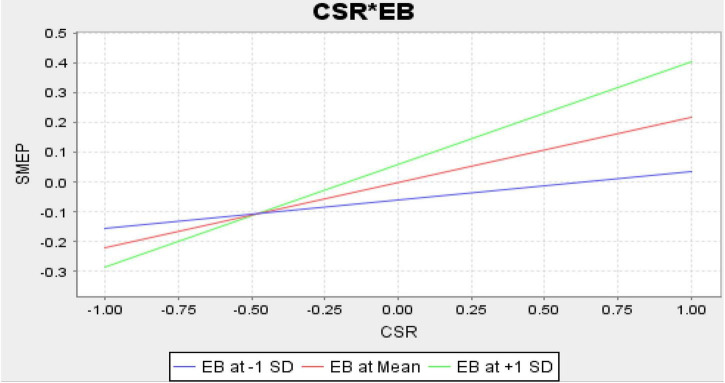
Corporate social responsibilities (CSR) × Employee behavior (EB).

**FIGURE 6:**
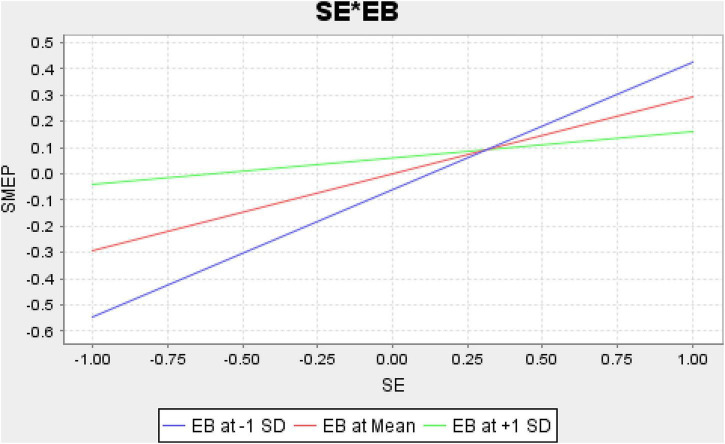
Social entrepreneurship (SE) × Employee behavior (EB).

**FIGURE 7:**
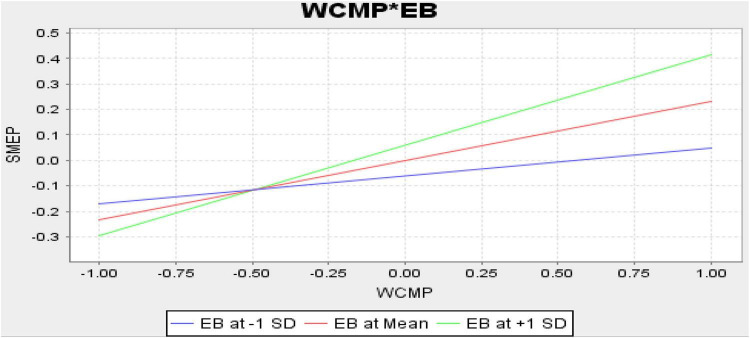
Working capital management practices (WCMP) × Employee behavior (EB).

Finally, the structural model assessment has been executed that shows the nexus among the variables. The results indicate that CSR, social entrepreneurship, and WCMP have a positive association with the performance of SMEs in Malaysia and accept H1, H2, and H3. Moreover, the results also indicate that EB positively moderates among the nexus of CSR, social entrepreneurship, WCMP, and performance of SMEs in Malaysia and accept H4, H5, and H6. These relationships are highlighted in Table 6. Figure 4 shows the graphical picture of structural model assessment. Moreover, Figure 5 (CSR*EB), Figure 6 (SE*EB), and Figure 7 (WCMP*EB) show the graphical representation of variables.

**TABLE 6:** Path analysis.

Relationships	Beta	S.D.	T Statistics	*P*-Values	L.L.	U.L.
CSR ->SMEP	0.220	0.082	2.685	0.004	0.059	0.344
CSR × EB ->SMEP	0.124	0.071	1.733	0.043	0.002	0.219
SE ->SMEP	0.295	0.063	4.647	0.000	0.201	0.408
SE × EB ->SMEP	−0.193	0.069	2.801	0.003	−0.305	−0.073
WCMP ->SMEP	0.234	0.065	3.610	0.000	0.116	0.344
WCMS × EB ->SMEP	0.123	0.067	1.836	0.035	−0.005	0.218

## Discussion

The outcomes of the present research have indicated that CSR has played a positive role in the performance of SMEs in Malaysia and these outcomes are in line with the outcomes of the Ionescu (2021b) who also examined that the effective implementation of best practices of CSR could increase the performance of the organization. In addition, the results also expose that social entrepreneurship has also played a positive role in the performance of SMEs in Malaysia and these results are matched with the results of May et al. (2021) who also expose that effective social entrepreneurship has increased the performance of the organization. Moreover, the results also expose that the WCMP have also put a positive impact on the performance of SMEs (Marakova et al., 2021). The impact of WCMP on the profitability of a business has been elaborated by Pais and Gama (2015). In addition, Wasiuzzaman (2015) also studies the profitability of the manufacturing industry by managing working capital. Networking benefits have been utilized as the proportion of profitability being unequivocally identified with the cash conversion cycle. The outcomes demonstrate that individual parts of the cash conversion cycle might be enhanced by proper capital management practices. Moreover, (Baker et al., 2019) elaborate on the firm soundness based on credit history maintained by credit rating agencies. Our study shows that both SMEs maintain stock books 100%. However, variations in maintaining debtor book, creditor book, cashbook, and computer for operations exist between them.

A study by Afrifa (2016) reports that the use of computers for maintaining the record reduced the cost of working by SMEs and it also helped in management decisions as well. The study shows that SMEs maintain 100% cashbook. Significant differences are exhibited between SMEs for cash budget preparations, credit selling, and credit purchasing and policy.

Ahmed and Streimikiene (2021) studied a couple of SMEs that had money overflow to put resources into attractive protections to produce more income. Disappointments in numerous enterprises might be linked to the lack of income. Most SMEs do not have financial plans. The vast majority of the entrepreneurs sell and buy on credit. Credit-based trading provides space during financial shocks. The study shows that differences exist between construction and manufacturing enterprises in stock review policy and insurance plans (Kovacova and Lewis, 2021). They also describe that most of the SMEs have no insurance cover. Lack of insurance describes no safeguard of inventory against fire and theft.

The results also expose that the EB has positive moderating among the nexus of CSR and performance of SMEs and this outcome is similar to the results of Manzoor et al. (2019) who found the positive role of EB on the firm performance when the CSR is effectively implemented in the organization. In addition, the outcomes also indicated that the EB has positive moderating among the links of social entrepreneurship and performance of SMEs, and this outcome is matched with the outcomes of Hopkins and Potcovaru (2021) who find the positive role of EB on the firm performance when the effective social entrepreneurship is effectively implemented in the organization. Finally, the results also find that the EB has positive moderating among the nexus of WCMP and performance of SMEs and this outcome is in line with the results of Singhania and Mehta (2017) who also found the positive role of EB on the firm performance when they effectively managed the working capital in the organization.

## Conclusion, Implications, and Limitations

Finally, the study has concluded that the SMEs of Malaysia have implemented effective CSR practices in the organization along with effective social entrepreneurship and effective management of working capital, that is, the reason for the high performance of SMEs in the country. In addition, the EB of SMEs has also played a positive role in the performance of SMEs in accordance with effective CSR practices, effective social entrepreneurship, and effective management of working capital. The main target of the project was to analyze WCMP by SMEs during corona days. The results show that SMEs have variations in their account maintenance. SMEs have less use of computer for their record keeping. On average, SMEs have stock review strategies along with stock insurance. This study has provided the guidelines to the policymakers that they should focus on developing the regulation related to CSR along with WCMP and EB that could increase the performance of SMEs. The present study has also provided the guidelines to the upcoming researcher while examining this area in future. This study contributes to the literature of SMEs’ performance in accordance with CSR, social entrepreneurship, and WCMP along with EB.

Finally, the present study has some limitations that guide future studies to be incorporated in their studies. The present study has taken EB as moderating variable and ignored the mediating impact on the model and suggested that future studies should incorporate this aspect in their studies. In addition, the ongoing study has also taken only three predictors such as CSR, social entrepreneurship, and WCMP and ignores the other factors that also affect the SMEs performance and recommended that future studies should also include the factors other than used by the present study in their analysis to predict the SMEs performance. Moreover, the current study has also examined the SMEs of Malaysia, ignored the other sector and countries, and suggested that future studies should add more sectors and countries in their studies. Finally, the present study has ignored the cross-country analysis and suggested to the upcoming studies that they should incorporate this point in their studies.

## Data Availability Statement

The raw data supporting the conclusions of this article will be made available by the authors, without undue reservation.

## Ethics Statement

Ethical review and approval was not required for the study on human participants in accordance with the local legislation and institutional requirements. Written informed consent for participation was not required for this study in accordance with the national legislation and the institutional requirements.

## Author Contributions

YL contributed to funding acquisition and data collection. IA contributed to the literature review. HR contributed to the conceptual development and proposition of hypotheses and introduction. AS contributed to proofreading and language editing. ER-A contributed to methodology and data analysis. EA-S contributed to the discussion, conclusion, and limitations. All authors contributed to the article and approved the submitted version. All authors listed have made a substantial, direct, and intellectual contribution to the work, and approved it for publication.

## Conflict of Interest

The authors declare that the research was conducted in the absence of any commercial or financial relationships that could be construed as a potential conflict of interest.

## Publisher’s Note

All claims expressed in this article are solely those of the authors and do not necessarily represent those of their affiliated organizations, or those of the publisher, the editors and the reviewers. Any product that may be evaluated in this article, or claim that may be made by its manufacturer, is not guaranteed or endorsed by the publisher.
